# Supplementary figures and images for: Soft-metal(loid)s induce protein aggregation in Escherichia coli
Source: Front Microbiol. 2023 Nov 22;14:1281058. doi: 10.3389/fmicb.2023.1281058 (PMC10699150; doi:10.3389/fmicb.2023.1281058)

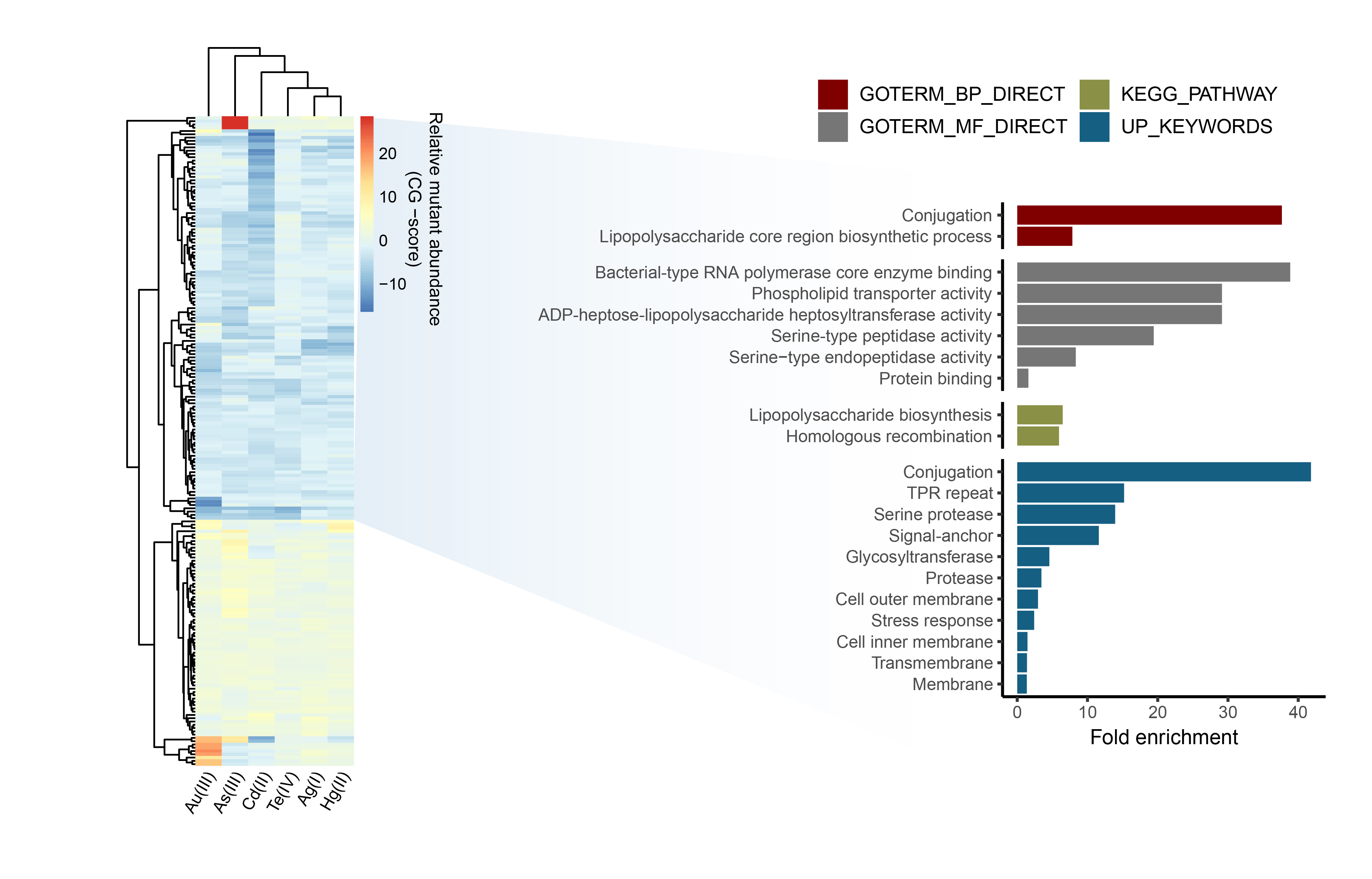

Supplement: Supplementary file 4 [file Image_1.JPEG]

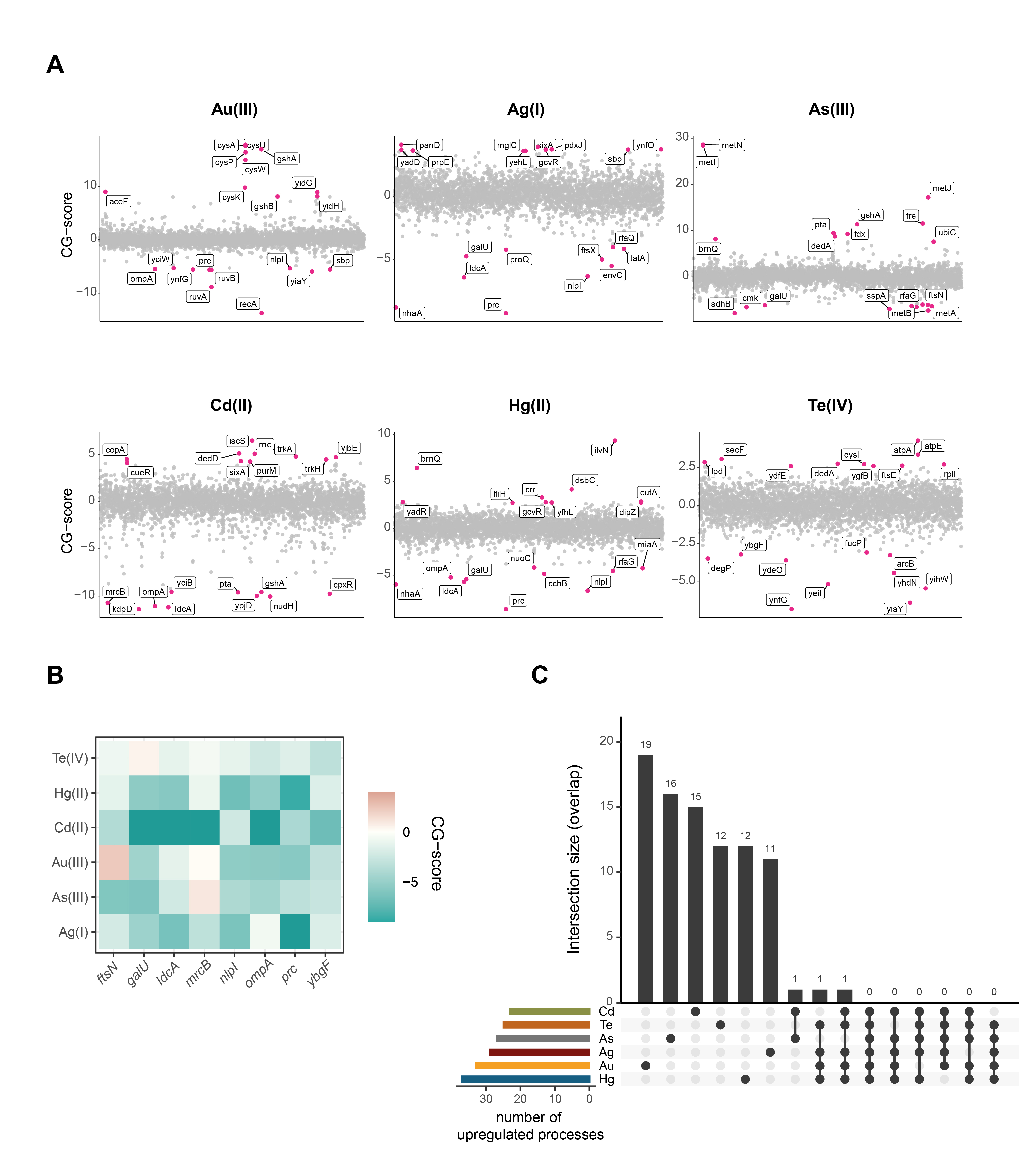

Supplement: Supplementary file 5 [file Image_2.JPEG]

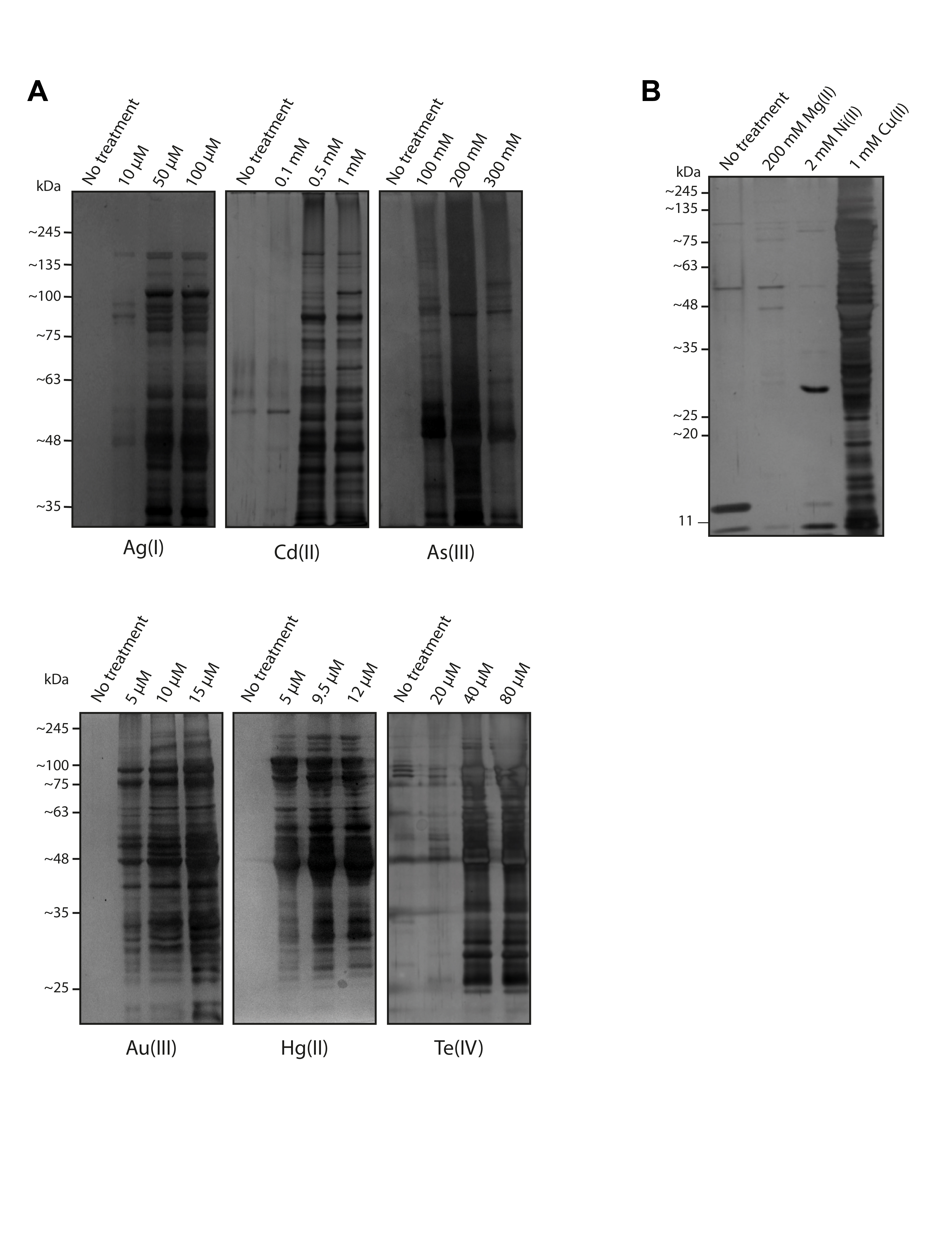

Supplement: Supplementary file 6 [file Image_3.JPEG]

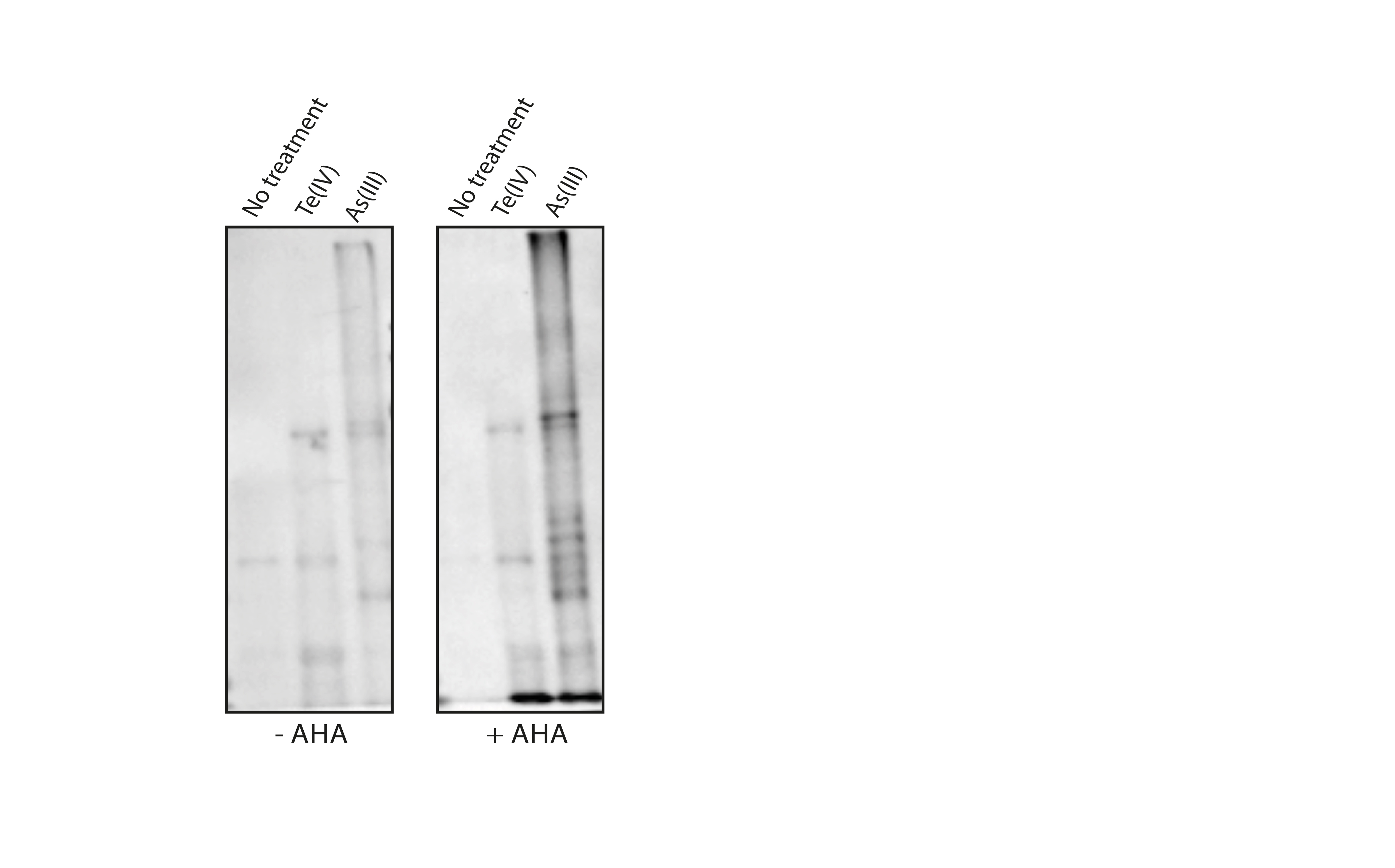

Supplement: Supplementary file 7 [file Image_4.JPEG]

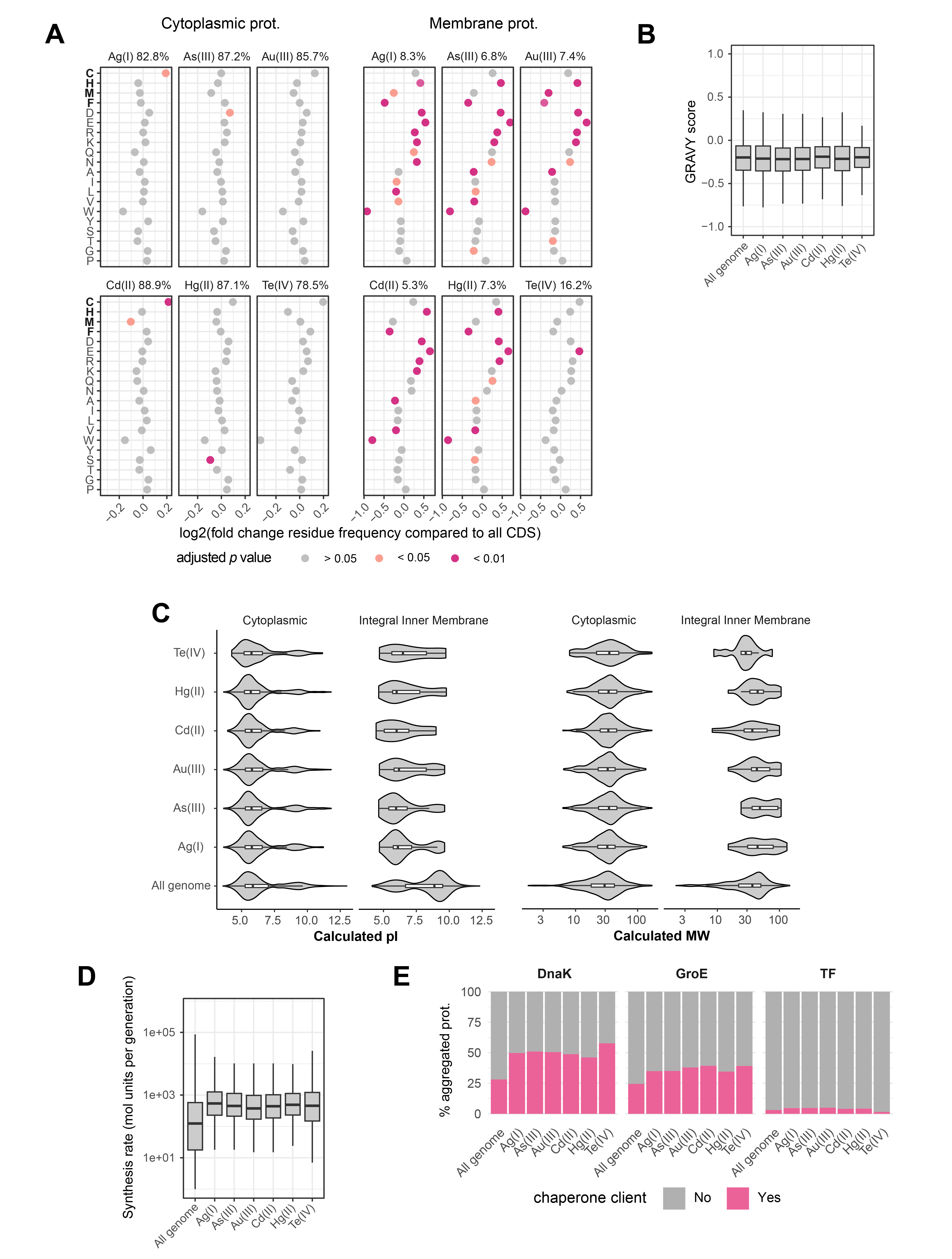

Supplement: Supplementary file 8 [file Image_5.JPEG]

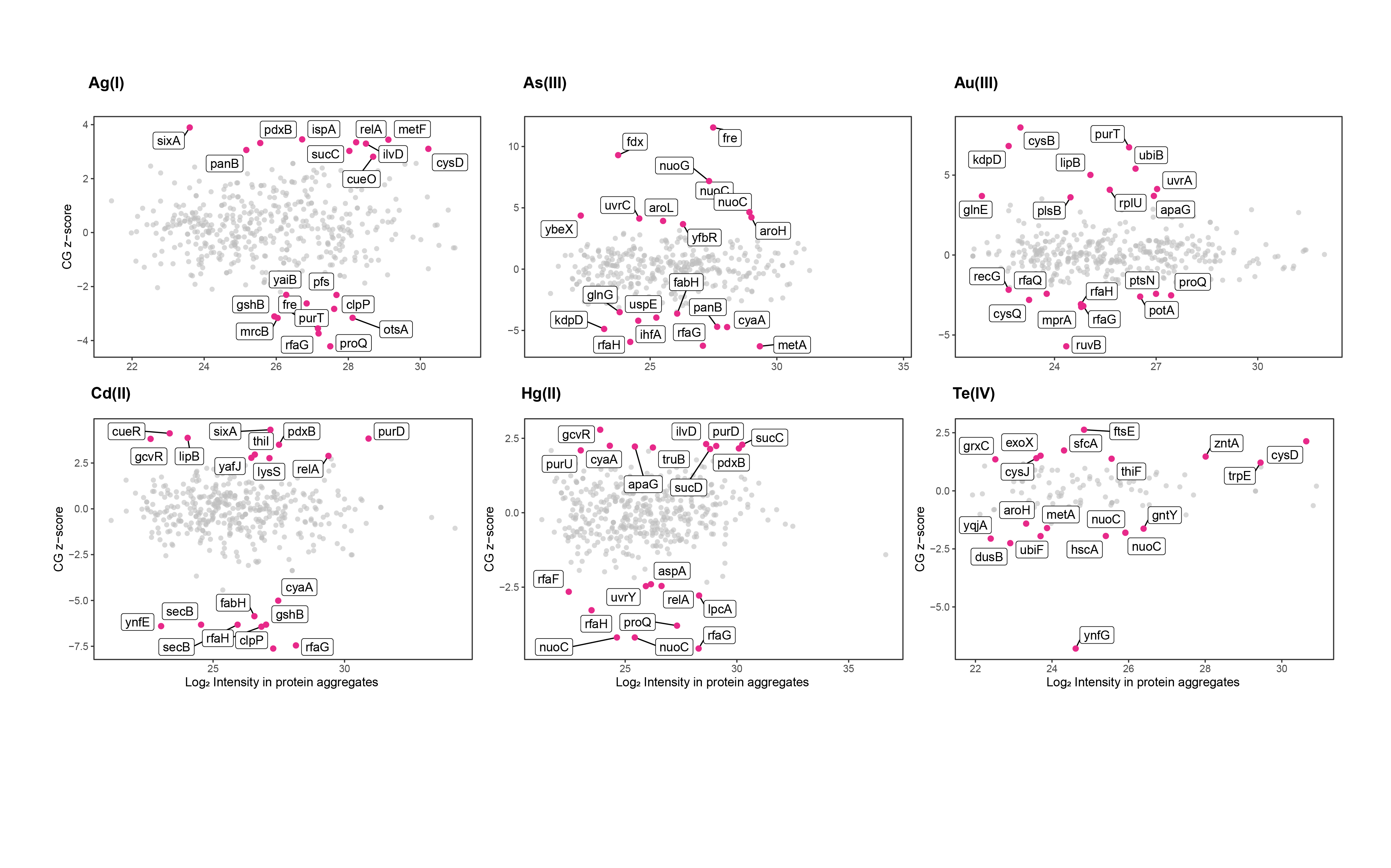

Supplement: Supplementary file 9 [file Image_6.JPEG]
